# Outcomes Following Closure of Secundum Atrial Septal Defect in Children ≤ 15 kg in a French Tertiary Centre

**DOI:** 10.3390/jcm13010198

**Published:** 2023-12-29

**Authors:** Claire-Marie Pilard, Olivier Villemain, Gérald Laforest, François Roubertie, Jean-Benoit Thambo, Zakaria Jalal

**Affiliations:** 1 Department of Paediatric and Adult Congenital Cardiology, Bordeaux University Hospital, 36000 Pessac, France; olivier.villemain@chu-bordeaux.fr (O.V.); gerald.laforest@chu-reunion.fr (G.L.); francois.roubertie@chu-bordeaux.fr (F.R.); jean-benoit.thambo@chu-bordeaux.fr (J.-B.T.); zakaria.jalal@chu-bordeaux.fr (Z.J.); 2 Plateforme Technologique d’Innovation Biomédicale, Centre de Recherche Cardio-Thoracique de Bordeaux, Bordeaux University, INSERM U1045, 33600 Pessac, France; 3 Institut Hospitalo-Universitaire Liryc, Electrophysiology and Heart Modeling Institute, Fondation Bordeaux Université, 33600 Pessac, France; 4 Institut National de la Santé et de la Recherche Médicale, Centre de Recherche Cardio-Thoracique de Bordeaux, INSERM U1045, 33600 Pessac, France

**Keywords:** secundum atrial septal defect, surgical closure, transcatheter closure, children, perioperative outcomes

## Abstract

Secundum atrial septal defects (sASDs) are common congenital cardiac defects mostly treated using a transcatheter approach. However, small children (<15 kg) are still undergoing surgical sASD closure in many centres. Although both options have been proved to have excellent results in children, comparative data of the two techniques are missing for patients ≤ 15 kg. The medical records of children ≤ 15 kg who underwent sASD surgical (group A) and transcatheter (group B) closure between 2010 and 2023 were reviewed retrospectively. Twenty-five children in group A and twenty-two in group B were included (mean weight 8.9 kg in group A and 10.3 kg in group B). The main indications for closure were right heart enlargement and failure to thrive. Major complications occurred in two patients in group A and none in group B. Minor complications occurred in eight patients in group A and one in group B. At last follow-up, symptoms resolved completely or improved significantly for all infants, with the exception of failure to thrive in the sub-population of children with extra-cardiac comorbidities. sASD closure can be performed safely in symptomatic infants ≤ 15 kg, even in the presence of comorbidity, and should not be postponed. However, in patients with extra-cardiac comorbidities, the only indication of growth retardation must be carefully evaluated.

## 1. Introduction

Atrial septal defect (ASD) is a common congenital heart defect with an incidence of 7%, and secundum ASDs (sASDs) represent 75% of atrial defects [1]. Atrial left-to-right shunting is usually well tolerated, and the majority of patients with isolated sASD remain asymptomatic in infancy and childhood. In addition, there is a significant spontaneous closure rate for small to moderate sASD [2]. However, sASD closure may be beneficial for a minority of infants and small children with isolated sASD at a much younger age as they present symptoms such as recurrent respiratory infections, heart failure, or failure to thrive. Historically, surgery has been considered to be the gold standard for sASD closure for symptomatic children under 2 years of age and ≤15 kg, with good long-term postoperative results [3,4]. However, since the first catheter-based closure of sASD described by King and Mills in 1976 [5], the procedure has undergone tremendous advancements and refinements of device designs and is considered safe and effective for older children and adults. Concerning younger children, common practice and recommendations suggest a body weight of more than 15 kg to allow for safe percutaneous closure [6,7]. Therefore, transcatheter sASD closure is usually postponed to the age of 4–6 years and only limited feasibility, safety, and effectiveness data have been reported for the transcatheter closure of sASD in infants weighing less than 15 kg.

In this work, we report our single-centre experience of sASD closure in children with a bodyweight ≤ 15 kg. The aim was to evaluate the outcomes and clinical benefits of sASD closure in this population, regardless of the technical approach (either surgical or percutaneous).

## 2. Materials and Methods

### 2.1. Study Design

We performed a monocentric, observational retrospective cohort study that was approved by the institutional review board of our centre. Informed consent was obtained from each of the study participants’ parents or legal guardian.

### 2.2. Study Population

Infants and children ≤ 15 kg who underwent surgical or transcatheter closure of an sASD between January 2000 and November 2023 in a French tertiary centre (Bordeaux University Hospital) were considered for inclusion. Syndromic and preterm birth patients were included in the study irrespective of the severity of their extra-cardiac impairment.

### 2.3. Procedures

For patient who underwent surgical closure, standard sASD repair was performed under general endotracheal anaesthesia, cardiopulmonary bypass, and anterograde cardioplegia. The right atrium was opened after a sternotomy or right posterior thoracotomy. The sASD was closed either by direct suture or using a pericardial or Goretex patch (W.L. Gore Associates, Flagstaff, AZ, USA). Patients recovered in the intensive care unit, where they were extubated and then transferred to the general care unit for further convalescence.

In group B, all the patients underwent cardiac catheterization under general anaesthesia with endotracheal intubation and with continuous transthoracic echocardiographic monitoring. In order to shorten procedural duration in these potentially vulnerable patients, complete haemodynamic workup (Qp:Qs, pulmonary pressures, pulmonary vascular resistances) was limited to patients with echographic signs of pulmonary hypertension (tricuspid regurgitation peak velocity > 2.5 m/s and right ventricular systolic pressure > 35 mmHg). All procedures were performed with the Amplatzer septal occluder (Abbott, Chicago, IL, USA). The device size reported is the size of the central portion (manufacturer sizing). The type and size of device were chosen at the discretion and based on the experience of the operator. The implantation technique was not standardized, but the technical recommendations of the manufacturer were generally followed. Operators were free to choose technical variations thought necessary to optimize results. Patients recovered in the intensive care unit, where they were extubated and spent the night in the hospital before discharge. Aspirin was orally administered for 6 months after the procedure at a dose of 3 to 5 mg/kg/day. All patients were instructed about infective endocarditis prophylaxis for a total of six months after device placement.

ECG and transthoracic echocardiography (TTE) were recorded before each patient’s discharge.

### 2.4. Data Collection

A computerized institutional database was used to screen patients for inclusion. Data were prospectively collected at the time of closure and every available follow-up appointment. Recorded data included demographic characteristics, growth parameters including height and weight percentiles, pre or per-procedure hemodynamic measurements (Qp:Qs ratio), type and timing of closure, indication for closure and presence of additional comorbidities including prematurity (defined as birth < 37 weeks of gestation), and chromosomal abnormalities or other comorbidities known to affect growth. Weight measurements were converted to weight-for-age percentiles following the World Health Organization’s (WHO) charts according to the Centers for Disease Control and Prevention [8]. Specific weight-for-age charts were used for the infants with Down and Turner syndromes. For premature infants, age was corrected according to prematurity. Polypnea was evaluated according to normal ranges of respiratory rates previously published by Fleming et al. [9]. Recurrent respiratory infections were defined by hospitalization and/or radiographic documentation and use of antibiotics. The degrees of right heart enlargement (RHE) were based on the official echocardiogram reports and determined by various, experienced cardiologists’ interpretations according to the recommendations of the American Society of Echocardiography [10]. sASD diameter was measured during echocardiography in subcostal, apical four chambers, and parasternal short-axis views in order to determine the sASD location, number, largest diameter and adequacy of its rims, and the presence of associating cardiac comorbidities. sASD was large if the diameter ASD/body surface area ratio was >20 mm/m^2^, as previously described by our team [11].

### 2.5. Follow-Up Evaluation

Due to their congenital cardiopathy, all children had a follow-up investigation before closure and then at 1 month, 6 months, and once a year after closure. They had a complete physical examination and echocardiography. Residual shunt was diagnosed if colour Doppler flow mapping showed a left-to-right or right-to-left shunt across the atrial septum. 

### 2.6. Statistical Analysis

Descriptive data are presented as mean +/− standard deviation (SD) and median (range) as appropriate. Proportions are expressed as percentages. For continuous data, a t-test was used to assess differences between the two groups (Mann–Whitney when comparing 2 groups with no-Gaussian distribution). Differences between weight centiles in sub-populations were tested using multiple Wilcoxon tests. For categorical data, Fisher’s exact test was used to analyse differences between the groups. *p* < 0.05 was considered to be statistically significant. Analyses were performed using GraphPad Prism version 9.00 for Windows (GraphPad Software, San Diego, CA, USA).

## 3. Results

### 3.1. Population Baseline Characteristics

As summarized in Table 1, statistically significant differences were observed in certain demographic data or preoperative clinical characteristics between the two groups. 

The mean age of patients was 18.1 ± 8.8 and 31.7 ± 14.5 months for group A and group B (*p* < 0.0001), respectively. The mean body weight was 8.9 ± 2.6 kg in group A and 10.3 ± 2.2 kg in group B (*p* = 0.0553). The proportion of children with a right heart enlargement (RHE) was significantly higher in group A than in group B (100% versus 82%, *p* = 0.041).

For children born prematurely, the birth terms were 30 and 35 (group A) and 29 and 31 (group B) weeks of amenorrhea. They had no complications related to their prematurity.

Genetic disorders were Down syndrome (one patient in group A), Noonan syndrome (two patients in group A), CHARGE syndrome (one patient in group A), a chromosomal anomaly such as partial trisomy 3p and monosomy 18q (one patient in group A), and mosaic Turner syndrome (one patient in group B).

Patients with polymalformative syndrome in group A had a large omphalocele and a pulmonary hypoplasia complicated by pulmonary hypertension. In group B, the first patient had a neurodevelopmental delay associated with deafness and strabismus and the second had severe bilateral congenital glaucoma, profound deafness, and congenital talipes equinovarus.

### 3.2. Anatomical and Hemodynamic Data (Table 2)

Invasive hemodynamic assessment performed before sASD closure showed that there was the same proportion of children (50%) with a mean pulmonary arterial pressure (mPAP) > 20 mmHg in both groups.

sASD size was significantly larger in group A than in group B (16.6 ± 4.1 mm versus 13.4 ± 5.1 mm, *p* = 0.0259). The number of large sASD (>20 mm/m^2^) was significantly higher in group A than in group B (100% versus 68%, *p* = 0.0027). Deficient posterior rim was significantly higher in group A than in group B (100% versus 25%, *p* = 0.0015), while deficient retroaortic rim was only present in patients who benefited from a transcatheter closure. 

**Table 2:** Anatomical and hemodynamic data.

	A	B	*p*
Catheterization	16% (4/25)	9% (2/22)	NS
Qp:Qs	2 ± 1 (2)	-	
>1.5	75% (3/4)	-	
mPAP (mmHg)	22.5 ± 5.2 (16–30)	20 ± 7.6 (12–28)	
>20 mmHg	50% (2/4)	50% (1/2)	
PVR (Wood units/m^2^)	1.5 ± 0.7 (0.8–2.4)	-	
sASD size (mm)	16.6 ± 4.1 (10–25)	13.4 ± 5.1 (5–22)	0.0259
>20 mm/m^2^	100% (25/25)	68% (15/22)	0.0027
Multiple sASD	16% (4/25)	27% (6/22)	0.4796
sASD/BSA (mm/m^2^)	40.2 ± 12.7 (25.5–75.9)	28.2 ± 11 (11.1–53.7)	0.0046
sASD/weight (mm/kg)	2.05 ± 0.8 (1.3–4.3)	1.4 ± 0.6 (0.5–2.75)	0.0032
Deficient septal rim	40% (10/25)	36% (8/22)	>0.9999
Posterior rim	100% (10/10)	25% (2/8)	0.0015
Retroaortic	-	75% (6/8)	0.0015

A = surgery group, sASD = secundum atrial septal defect, B = transcatheter group, BSA = body surface area, mPAP = mean pulmonary artery pressure, NS = not significant, PVR = pulmonary vascular resistance, Qp = pulmonary flow, Qs = systemic flow. Continuous data are presented as mean ± standard deviation (range), and categorical variables are presented as percentage (number).

### 3.3. Procedural Details (Table 3)

Table 3 lists the procedural detail. Procedures in both groups were performed under general anaesthesia with endotracheal intubation. All patients survived the procedure. One patient with severe pulmonary artery hypertension had a fenestrated sASD patch in group A, and another one had a manually fenestrated device in group B. Successful closure was obtained in all infants in group A and in 95.5% (21/22) of infants in group B. Transcatheter closure failed in one child (28 months and 8 kg) in group B for anatomical reasons (posterior rim deficiency). In group B, the initially implanted device, which was too large, was replaced by a second smaller one in two patients.

In group B, the diameter of sASD ranged from 5 to 22 mm (Table 2), whereas the size of the implanted occluder ranged from 8 to 26 mm (16 ± 5 mm). The ratio of the applied device size to body weight reached 1.6 ± 0.6, and 59% of patients had a large occluder (device/weight ratio ≥ 1.5).

**Table 3:** Procedural details.

	A	B
Aortic cross clamp time (min)	25 ± 8.6 (12–55)	-
Cardiopulmonary bypass (min)	56 ± 17.9 (20–106)	-
Fluoroscopy time (min)	-	5.1 ± 3.1 (3.7–19.2)
Surgical approach		
Sternotomy	64% (16/25)	-
Lateral thoracotomy	36% (9/25)	-
Vascular approach		
Right femoral vein	-	100% (22/22)
Surgical closure		
Direct suture	16% (4/25)	-
Patch	84% (21/25)	-
Heterologous	71.4% (15/21)	-
Autologous	4.8% (1/21)	-
Goretex	23.8% (5/21)	-
Including fenestrated	4.8% (1/21)	-
Transcatheter closure		-
Procedural failure	-	4.5% (1/22)
Venous introducer size	-	6.45 ± 1.1 (5–10)
Device size	-	16 ± 5 (8–26)
Device/weight ratio (mm/kg)	-	1.6 ± 0.6 (0.7–2.5)
Device/weight ratio ≥ 1.5	-	59% (13/22)

A = surgery group, B = transcatheter group. Continuous data are presented as mean ± standard deviation (range), and categorical variables are presented as percentage (number).

### 3.4. Immediate Postoperative Data (Table 4)

Length of stay was significantly shorter in group B than in group A (2.6 ± 0.5 versus 9.4 ± 2.5, *p* < 0.0001), and no patient stayed in intensive care unit in group B. In group A, 56% needed a respiratory support as a relay to mechanical ventilation, and 84% received an inotropic support (milrinone) during 2 ± 1.1 days.

Trivial residual shunts were documented immediately after the procedure in two and three patients in groups A and B (8% versus 13.7%, *p* = 0.6536), respectively.

Death, erosion, tamponade, cardiac perforation, atrioventricular valve distortion, endocarditis, thromboembolism, or permanent rhythm disturbances were absent in both groups. The rate of periprocedural complications was significantly higher in group A than in group B (40% versus 4.5%, *p* = 0.0054) due to an increased rate of minor complications in group A than in group B (32% versus 4.5%, *p* = 0.0252) (Table 4). In group A, two patients had major complications: one patient had developed a sinus node dysfunction treated with a pacemaker in 20 days, and one patient had a chylothorax complicated with a respiratory tract infection. In the same group, eight patients experienced minor in-hospital complications, including pericardial effusion treated with medication and documented infections requiring treatment. One patient had a thrombopenia treated with platelets transfusion. Two patients had an episode of supraventricular tachycardia and were treated with amiodarone. In group B, transient arrhythmia (complete atrio-ventricular block) occurred in one patient during the procedure and resolved totally after device removal. A 4 mm smaller occlude was subsequently implanted without complication. There was no major complication in group B (Table 4).

**Table 4:** Immediate postoperative data.

	A	B	*p*
Length of stay (days)	9.4 ± 2.5 (6–18)	2.6 ± 0.5 (2–3)	<0.0001
Including ICU (days)	5 ± 2 (2–10)	-	<0.0001
Inotropic support	84% (21/25)	-	<0.0001
Duration (days)	2 ± 1.1 (1–5)	-	<0.0001
Respiratory support	56% (14/25)	-	<0.0001
Duration (days)	2.3 ± 1.6 (1–6)	-	<0.0001
Residual shunt	8% (2/25)	13.7% (3/22)	0.6536
Periprocedural complications	40% (10/25)	4.5% (1/22)	0.0054
Major complications	8% (2/25)	-	0.4912
Chylothorax	4% (1/25)	-	>0.9999
Permanent arrhythmia	4% (1/25)	-	>0.9999
Minor complications	32% (8/25)	4.5% (1/22)	0.0252
Pericardial effusion	4% (1/25)	-	>0.9999
Documented infection	16% (4/25)	-	0.1119
Transient arrhythmia	8% (2/25)	4.5% (1/22)	0.6115
Thrombopenia	4% (1/25)	-	>0.9999

A = surgery group, B = transcatheter group, ICU = intensive care unit. Continuous data are presented as mean ± standard deviation (range), and categorical variables are presented as percentage (number).

### 3.5. Follow-Up Outcomes

After a median follow-up duration of 6 months (mean 20.9 ± 22.7 months; range 1–87 months) in group A and 6 months (mean 8.1 ± 5.9 months; range 1–24 months) in group B, no death, thromboembolic events, aortic erosion, occlude dislodged, nor other major complications were observed.

**Figure 1:**
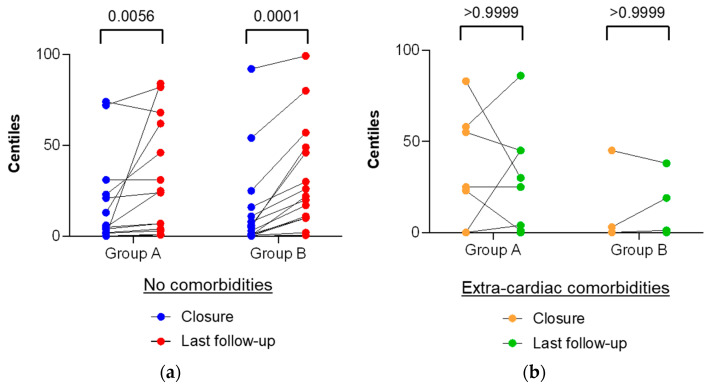
Weight improvement after secundum ASD closure. (**a**) Weight improvement expressed as percentiles in the subpopulation of children without extra-cardiac comorbidities. Group A with n = 14 children and group B with *n* = 15 children with available weight values at last follow-up; (**b**) weight improvements expressed as percentiles in the subpopulation of children with extra-cardiac comorbidities. Group A with n = 8 children and group B with *n* = 4 children with available weight values at last follow-up. Weight measurements were converted to weight-for-age percentiles following the World Health Organization’s (WHO) charts according to the Centers for Disease Control and Prevention. Specific weight-for-age charts were used for the infants with Down and Turner syndromes. For premature infants, age was corrected according to prematurity. A = surgery group, B = transcatheter group.

To date, symptoms either resolved completely or improved significantly for all symptomatic infants, with the exception of failure to thrive in the subpopulation of children with extra-cardiac comorbidities in both groups. Indeed, among children with available follow-up data (22/25 in group A and 19/22 in group B), weight percentiles improvement after sASD closure was significantly better in the subpopulation of infants without extra-cardiac comorbidities in both groups than in the subpopulation of infants with extra-cardiac comorbidities (Figure 1).

In both groups, among children with immediate postoperative trivial residual shunt, none exhibited any residual shunt at the 6-month follow-up evaluation. One patient in each group had pulmonary hypertension before sASD closure and had beneficiated from a fenestrated patch for the patient in group A and a manually fenestrated device for the patient in group B. At the 7-month follow-up evaluation, right ventricular systolic pressure normalized at echocardiography, and there was no residual intra-device shunt for both patients. 

Right heart enlargement was still observed at the end of the follow-up evaluation in three patients in group A and one in group B (12% versus 4.5%, *p* = 0.6115). 

## 4. Discussion

In this study focusing on the clinical benefits of sASD closure in children < 15 kg, we showed that (1) both transcatheter and surgical approaches were safe and effective techniques for sASD closure, and (2) preterm and genetic disorder patients with growth retardation did not significantly improved their clinical condition following closure.

Moreover, our study has demonstrated the benefits of transcatheter closure in terms of lower complication rates and mean hospital stay. However, surgery still has a place for large and complex defects closure.

### 4.1. Comparison in Efficacy

The success rates in both groups and immediate residual shunt after surgical closure are similar to those of other studies [11,12,13,14,15]. Our rate of immediate residual shunt in the transcatheter group was higher compared to the previous nationwide cohort study of sASD closure in children [11] and other studies in children ≤ 15 kg [16,17,18,19,20,21,22,23]. However, all residual shunts were classified as trivial or small, and no residual shunt was found at the 6-month follow-up evaluation. In addition, apart from growth restriction in the sub-group of children with extra-cardiac comorbidities, no children were symptomatic in both groups at the end of the follow-up.

### 4.2. Comparison of Safety

There was no mortality in both groups. This is in accordance with previous reports in the current era [11,12,13,14,15,16,17,18,19,20,21,22,23,24,25,26]. There was a significant higher rate of periprocedural complications between the two groups (40% in group A versus 4.5% in group B, *p* = 0.0054) due to a significantly higher rate of minor complications in group A compared to group B (Table 4). This is in line with the available literature. Indeed, Ooi et al. reported a significant higher complication rate in 3159 children who underwent surgical sASD closure compared with 4606 children who had a percutaneous sASD closure (19.8% versus 3.7%; odds ratio: 6.66; *p* < 0.0001) [16]. Our results about the surgical closure of sASD are also consistent with the available literature in terms of complications. The total rate of reported complications in various studies ranges from 31 to 68%, with a proportion of major complications varying from 4 to 16% [12,13,14,15,16,25,26,27,28,29]. In our series, the rate of major complications is 8%, which is similar to that of previously published studies, including a meta-analysis with 1270 patients that reported a 6.8% rate of adverse events [14], although our patients were smaller in terms of age and weight. No major complication occurred in the transcatheter group before discharge and during follow-up, which is in line with other studies [11,12,13,14,15,16,17,18,19,20,21,22,23,24]. One patient had a per-procedural transient atrio-ventricular block, which resolved spontaneously after the device was resized. 

Finally, in our study, length of stay was significantly longer in the surgical sASD closure group compared with the transcatheter group (9.4 ± 2.5 versus 2.4 ± 0.5; *p* < 0.0001) (Table 4), which is in line with previous reports [12,13,14,15,16]. This can be explained by the high rate of children with comorbidities who required longer postoperative care and recovery. In fact, the extracorporeal circulation and thoracotomy required for surgery are not without consequences in premature infants with bronchopulmonary dysplasia or in children with Down syndrome at risk of pulmonary hypertension. Hence, transcatheter sASD closure may be an efficient and safe option to avoid the detrimental effects of cardiopulmonary bypass in this specific population.

### 4.3. Subgroup Analysis: Additional Comorbidities and Weight Improvement

Poor growth or failure to thrive, defined as a plateau or a decline in growth velocity, is one of the reasons why these patients are commonly sent for surgical closure; this accounted for 60% of children in group A and 45% in group B (Table 1). Previous studies have shown that children with sASD may experience improved growth after sASD closure [25,26,27,28,29], although the mechanism of growth acceleration after sASD repair is unclear. Among children with available follow-up data (22/25 in group A and 19/22 in group B), twelve (54%) patients in group A and thirteen (74%) patients in group B showed increased weight percentile gain after closure, and we showed that weight percentile improvement was significantly higher in patients without genetic abnormality or prematurity compared to patients with extra-cardiac comorbidities (group A: *p* = 0.0056 versus > 0.9999; group B: *p* = 0.0001 versus > 0.9999) (Figure 1). This result suggests that an early sASD closure should be considered in symptomatic children without extra-cardiac comorbidities before reaching 15 kg to help them to thrive, but in children with genetic disorders or prematurity, origin of growth retardation is complex, and early closure does not seem to improve growth. It may be safer to delay the sASD closure and propose a device closure to these children. Prior studies have demonstrated growth improvements after sASD closure in children with an initial growth percentile ≤ 5th percentile [30] or ≤ 16th percentile [31]. However, the populations in these studies were older (between 3.4 and 6 years) and larger. Moreover, Rhee et al. excluded children with extra-cardiac comorbidities known to have an intrinsic profound impact on growth.

### 4.4. Study Limitations

This is a retrospective study with a small population size in both groups. The disparity of age is explained by the fact that surgery has long been the gold standard in our centre, with surgeons having considerable experience in young children. Percutaneous closure before 15 kg has only become standard practice in recent years thanks to improvements in the available techniques. So, initially, first catheterizations were performed on older and larger children. The age disparity explains the non-significant difference in body weight (Table 1).

Another limitation is the short follow-up time. Because of our network organization, medium- and long-term follow-ups are carried out by cardiologists close to home, and data collection is not protocolized.

Finally, in group A, 32% (8/25) of children had an extra-cardiac comorbidity known to affect growth, such as prematurity and chromosomal abnormalities, but disease-specific growth charts are only available for Down and Turner syndromes and prematurity. Therefore, failure to thrive in children with polymalformative syndrome or chromosomal abnormalities other than Down syndrome may have been slightly overestimated. Nevertheless, the same growth curve was used throughout the study for each child, so the bias was the same for each weight assessment.

## 5. Conclusions

In this study focusing on the clinical benefits of sASD closure in children < 15 kg, we showed that (1) both transcatheter and surgical approaches were safe and effective techniques for sASD closure, and (2) preterm and genetic disorder patients with growth retardation did not significantly improve their clinical condition following closure.

Moreover, our study has demonstrated the benefits of transcatheter closure in terms of lower complication rates and mean hospital stay. However, surgery still has a place for the closure of large and complex defects.

Therefore, defect closure should not be postponed to a later age in patients who have symptoms that can be attributed to left-to-right shunt, and transcatheter closure seems to be a safe and effective alternative treatment for sASD in this population.

In preterm children and patients with genetic disorders, the only indication of growth retardation for early closure must be carefully evaluated because growth improvement seems to be lower in this specific subgroup. These comorbidities are potential confounding variables that are likely to have a profound impact on growth and may serve as the foundation for a future study in order to better delineate if this particular subset of patients could also benefit from the early repair of their sASD.

## Figures and Tables

**Table 1:** Comparison of baseline characteristics of both groups.

Characteristics	A	B	*p*
Number of patients	25	22	
Age (months)	18.1 ± 8.8 (4–33)	31.7 ± 14.5 (8–54)	0.0003
Female	64% (16/25)	50% (11/22)	0.386
Weight (kg)	8.9 ± 2.6 (4.1–14)	10.3 ± 2.2 (6.3–14)	0.0553
Comorbidities	40% (10/25)	27% (6/22)	0.5381
Non-cardiac			
Prematurity	8% (2/25)	9% (2/22)	-
Genetic disorders	20% (5/25)	4.5% (1/22)	-
Down syndrome	4% (1/25)	-	-
Noonan syndrome	8% (2/25)	-	-
Other	8% (2/25)	4.5% (1/22)	-
Polymalformative syndrome	4% (1/25)	9% (2/22)	-
Cardiac			
Pulmonary stenosis	12% (3/25)	9% (2/22)	-
PDA	24% (6/25)	-	-
Aortic coarctation	-	4.5% (1/22)	-
Indications for sASD closure			
RHE	100% (25/25)	82% (18/22)	0.041
Failure to thrive	60% (15/25)	45% (10/22)	0.387
Polypnea	16% (4/25)	4.5% (1/22)	0.3525
Recurrent respiratory infections	20% (5/25)	-	0.4227
Pulmonary hypertension	8% (2/25)	-	>0.9999

A = surgery group, sASD = secundum atrial septal defect, B = transcatheter group, PDA = patent ductus arteriosus, RHE = right heart enlargement. Continuous data are presented as mean ± standard deviation (range), and categorical variables are presented as percentage (number).

## Data Availability

The data presented in this study are available on request from the corresponding author.
